# Quality of medicines in resource-limited settings: need for ethical guidance

**DOI:** 10.1080/11287462.2018.1522991

**Published:** 2018-09-18

**Authors:** Raffaella Ravinetto, Wim Pinxten, Lembit Rägo

**Affiliations:** a Public Health Department, Institute of Tropical Medicine, Antwerpen, Belgium; b Hasselt University, Hasselt, Belgium; c Council for International Organizations of Medical Sciences (CIOMS), Geneva, Switzerland

**Keywords:** Quality of medicines, ethics, beneficence, justice, developing countries

## Abstract

The quality of medicines is generally adequately assured by manufacturers and regulatory authorities for well-resourced settings, while the implementation of existing quality standards is challenged in many low- and middle-income countries. This situation of multiple pharmaceutical standards raises the question whether it could ever be ethically justified to compromise on the quality assurance of medicines depending on what individuals, communities, or societies can afford. In this paper, we contend that ethically, any unjustified exceptions to medicines’ quality assurance represents a violation of the principles of beneficence and non-maleficence. Exceptions are only acceptable in *exceptional* and *temporary* circumstances, if based on a meaningful quality risk assessment, guided by a rigorous ethical framework built on the principles of independence, technical competence, transparency, and accountability. We also discuss how such exceptional and temporary circumstances should be defined/justified. Finally, we propose that empirical bioethics should acknowledge the existence of these dilemmas in public health, and help to build a normative approach to dealing with them. Ideally, an international group of experts in quality assurance/regulatory affairs and health ethicists should be set up to take up this topic and formulate a Guide to Ethical Principles of Quality Assurance of Medical Products.

## Introduction

Since the birth of bioethics in the middle of the past century, ample attention has been devoted to the ethical aspects of biomedical research. Partly under the pressure of numerous scandals (Lenzer, 2011; McCarthy, 2015; Reverby, 2011) and critical incidents (Dally, 1998), drug development has become increasingly regulated, both in ethics codes (Belmont Report, 1979; Nuremberg Military Tribunal, 1996; World Medical Association, 2013) and in various international and national regulations. Good practices in preclinical and clinical research, pharmaceutical manufacturing and safety monitoring have been introduced. In addition, good practices of regulatory review of quality, safety and efficacy have been introduced, such as the Good Review Practices guidelines of the World Health Organization (WHO) and of the US Food and Drug Administration (FDA) (US Food and Drug Administration. Good Review Practices; World Health Organization, 2015). Finally, monitoring instruments, such as the Benchmarking of European medicines agencies (Heads of Medicines Agencies) or the WHO assessment of medicines regulatory systems (World Health Organization, Not dated), have been installed to maximize the follow-up to these good practices. Effective regulatory systems are increasingly recognized as an essential component of health system strengthening, and it is hoped that WHO guidance on Good Regulatory Practices (under finalization) will provide further means for establishing sound, affordable and effective regulation as an important part of health system strengthening, built on transparency, good governance and sound government policy-making (World Health Organization, October, 2016). At present, the quality of medicines can be adequately assured by regulatory authorities in well-resourced settings, where rare quality incidents result from occasional mistakes rather than from structural weaknesses. But the adequate implementation of the current standards is challenged in the resource-poor context of low- and middle-income countries (LMICs).

During recent years, there has been growing attention of bioethics to emerging global health-related challenges, such as the globalization of biomedical research (Lang & Siribaddana, 2012) and to the meaning and application of research ethics principles in the context of LMICs (Council for International Organizations of Medical Sciences (CIOMS) in collaboration with the World Health Organization (WHO), 2016; Emanuel, Wendler, Killen, & Grady, 2004; Glickman et al., 2009). However, the ethical discussion on the globalization of the pharmaceutical market and the quality of medicines in resource-poor settings is still in its infancy. Such an ethical discussion, however, is pertinent. The quality of medicines is currently unequal throughout the world, and particularly low in LMICs with weak legal and regulatory oversight of the pharmaceutical sector (t’Hoen, Hogerzeil, Quick, & Sillo, 2014). Many National Medicines Regulatory Authorities (NMRAs) still lack the resources and capacities to assure the quality of medicinal products manufactured, imported or circulating in their territory (World Health Organization, 2008, 2010), while pharmaceutical production and distribution have become increasingly global and complex since the 1990s (Caudron et al., 2008). Different authors have reported that poor-quality medicines are an important threat to individual and public health (Newton et al., 2011), highly prevalent in resource-limited settings (Almuzaini, Choonara, & Sammons, 2013; Johnston & Holt, 2014; McGinnis M. USAID-USP Media Reports on Medicine Quality: Focusing on USAID-assisted Countries. U.S, 2014; Nayyar, Breman, Newton, & Herrington, 2012; Supplement, 2015). Problematic products include “falsified” products, which stem from illegal activities (i.e. there is always a *deliberate or fraudulent* misrepresentation of their identity, composition or source) and “substandard” products, which may be unauthorized (e.g. illegally imported) or authorized by the NMRA, but fail to meet national and/or international quality standards due to poor practices that are not detected by the NMRAs (World Health Organization, 22–31 May, 2017). All are potentially harmful to the final users. For instance, under-dosing due to less active ingredient inside tablets or their poor bioavailability may result in (often undetected) therapeutic failure, while cross-contamination, accelerated degradation and lack of sterility may result in toxicity. In the case of anti-malarial medicines, under-dosing and poor bioavailability contributed to the emergence of resistances (Newton, Caillet, & Guerin, 2016). In addition, poor-quality medical products negatively affect patient and health-care worker confidence in generic medicines, even when fears are found to be unfounded by empirical evidence (Aivalli et al., 2018). More generally, they can also lead to loss of confidence in health care systems and governments. In today’s globalized pharmaceutical market, poor-quality medicinal products may be either manufactured locally, or imported from countries with variable regulatory enforcement such as India (Ravinetto, Dorlo, Caudron, & Prashanth, 2013), or imported from high-income countries. The latter is possible, since medicines manufactured for export only are not necessarily regulated to the same standard as those for domestic use (Caudron et al., 2008); for instance, compliance with Good Manufacturing Practices, which is essential but insufficient to address all the quality, safety and efficacy requirements, is often the only legal requirement for exported medicines.

While this situation of multiple pharmaceutical standards may be as undesirable as uncomfortable, it also raises the question whether it could ever be *ethically* justified to compromise on the quality assurance of medicines depending on what individuals, communities, or societies can afford. Positive experiences should also not be ignored when pursuing this reflection. In particular, the establishment in 2001 of the WHO Prequalification of Medicines Programme (PQP), in response to the need to assess the quality, safety and efficacy of generic medicines that were becoming increasingly available, especially for HIV/AIDS, was a major breakthrough (t’Hoen et al., 2014). It provided evidence that low price cannot be the primary criterion for procurement of medicines for LMICs, that quality standards can be raised, and that non-quality-assured medicines should not be purchased for poor people. As stated by t’Hoen and colleagues, “the WHO PQP has made it possible to believe that everyone in the world will have access to safe, effective, and affordable medicines” (t’Hoen et al., 2014).

## Nothing but the best is good enough? – technical and ethical issues in compromising in quality

Clear technical guidelines exist to define the quality assurance of any product or service, in order to prevent harm to the users, and this also applies to medicines. Quality assurance is a risk-management system, by which quality is “built” into a pharmaceutical product at every step of development and production, so to protect the final users from avoidable risks (WHO 2003; WHO 2015). Noteworthy, *safety* concerns should not be automatically equaled to *quality* concerns. Adverse reactions not linked to quality issues are intrinsic to the structural moiety of a medicine or vaccine, and patients who have exhausted or lack other therapeutic options, may be willing to take a medicine with a poor safety profile, because of the lack of alternatives. Conversely, as a rule there is an alternative to poor-quality medicines, that is replacing a product of unknown or poor quality with a quality-assured one. *⁠*But resource constraints may challenge the adoption and/or implementation of “full” quality standards. In many LMICs, this also applies to the quality assurance of medicines. From an ethical perspective, this raises the question whether the quality of medicines can ever be open to compromises under specific circumstances, or whether it is always unethical to go for anything but the best?

First, certain practices seem to be beyond ethical justification. If we have learned lessons from the historical research scandals that have been thoroughly debated in bioethics, manifest breaches of basic ethical principles, including the uncontrolled and avoidable exposure of individuals to *known* risks, can never be ethically justified. Any attempt to explore the ethical acceptability of trade-offs on quality can thus easily exclude such issues from their scope.

However, after eliminating clearly *unethical grounds* for compromising on quality assurance, there may be cases where no-treatment is a bigger threat to the life of patients than the threat from medicines that do not meet all quality standards. In such situations, a thorough quality risk assessment can lead to motivated exceptions until alternative products meeting all required standards become available, or the single-source product’s quality deficiencies have been eliminated, and this even in well-resourced settings. The *ethical imperative to ensure the quality of medicines* does not necessarily mean that meaningful quality risk assessment cannot result, in certain circumstances, in *exceptionally* and *temporarily* delivering medicines that do not meet all quality standards. However, any such decisions should be based on a quality risk assessment carried out prior to (political) decision-making, and should be guided by a rigorous ethical framework, built on the principles of full independence, technical competence, transparency, and accountability. For example, the WHO Expert Review Panel (ERP) is an independent advisory body of technical experts which assesses the potential quality risks of pharmaceutical products that do not meet all stringent quality requirements, based on transparent science-based criteria. The ERP provides advice to the Global Fund to Fight AIDS, Tuberculosis and Malaria (GFTAM) and other stakeholders for aiding decisions on time-limited procurement, by classifying products in one of four categories: no objection to time-limited procurement (only for products that are under assessment of PQP – Category 1 – or stringent regulatory authority – Category 2); objection to procurement but may be considered when there are no alternatives, and provided the benefit outweighs the risk of procuring a product which is not fully quality assured (Category 3); and objection to procurement (Category 4) (World Health Organization (WHO) Prequalification of Medicines Programme, 2012; World Health Organization Pre-qualification Team).

Notwithstanding that medicines are an essential commodity, and that *ethically the quality of medicines cannot be compromised except under exceptional circumstances*, we shall use this paper as a forum to discuss how such *circumstances* should be defined and justified.

## The predicament: structural flaws in assuring the quality of medicines in low- and middle-income countries

In many LMICs, the occurrence of substandard medical products is the rule rather than the exception. Shortcomings in the quality of medicines often cannot be attributed to *occasional* one-time mistakes, but are due to *structural* flaws, such as systemic poor or non-compliance with good practices. Reports abound in very recent literature. For instance, in Zambia in 2015, anaesthesia providers had noted various unpredictable adverse events and inadequate depth of anaesthesia, following the administration of one particular brand of the anaesthetic propofol. When three samples of the suspect product were sent for testing at an independent laboratory, it appeared that the active ingredient was underdosed to a serious extent (Mumphansa et al., 2017). When Antignac and colleagues prospectively assessed the quality of some medicines routinely used for cardiovascular diseases in ten Sub-Saharan African countries, they found that 16.3% of the tested samples were of poor quality (Antignac et al., 2017). The problems are not limited to sub-Saharan Africa. For instance, *post hoc* pharmacopoeial tests of omeprazole-containing medicines in Cambodia and Myanmar revealed high rates of non-compliance with adequate specifications, causing the premature release of the active ingredient after administration (Rahman et al., 2017). On a broader scale, in 2017 WHO estimated the observed failure rates of substandard and falsified medical products in LMICs at approximately 10.5% (World Health Organization, 2017).

We face three major problems: regulatory oversight, manufacturing practices, and purchasing practices.

First, regulatory oversight is inadequate in many LMICs, and in the absence of stringent regulatory oversight, manufacturing, procuring and distributing quality-assured medicines and other medical products of quality become a matter of *skills,* or of *choice,* or of *market demand,* rather than a duty. When it comes to a matter of *skills*, quality will depend on manufactures’ and distributors’ own ability to work in compliance with the WHO Good Manufacturing Practices and other essential technical standards. When it comes to a matter of *choice*, quality will depend on manufactures’ and distributors’ corporate policy; for instance, a local distributor may focus on medicines approved by Stringent Regulatory Authorities because it tries to serve the “niche” of clients who ask such products, or because it tries to differentiate its own offer from competitors (Van Assche et al., 2018). When it comes to a matter of *market*, quality will depend on the incentives to quality; for instance, many manufacturers of medicines for HIV/AIDS, malaria and tuberculosis have upgraded their standards to those of the WHO PQP (t’Hoen et al., 2014), since WHO pre-qualification is an essential requirement for the tenders of major purchasers such as the Global Fund, United Nations agencies and international Non-governmental Organizations.

Second, poor manufacturing practices hamper the quality of medicines manufactured or imported in insufficiently regulated countries, since under-resourced regulatory authorities may fail to detect poor practices upfront (i.e. through stringent inspections at the manufacturing sites, and stringent assessment of products’ dossiers). As a consequence, they will fail to *prevent* such poor manufacturing practices and their consequences. For instance, contaminated anti-cough and anti-arrhythmic medicines were identified, respectively in Panama and in Pakistan, only *after* they had caused clusters of serious adverse events and deaths (Arie, 2012; Danielle Rentz et al., 2008).

Third, the hospitals, private wholesalers, pharmacies, nongovernmental organizations, etc. are either unaware of these risks (e.g. the in-house technical expertise to assess the risks and benefits may be insufficient or inadequate), or aware but not ready to set up alternative procurement channels. Purchasers who are aware of the inherent risks may end up compromising between availability/accessibility and assured quality, for reasons that may include lack of financial or logistic resources, lack of institutional willingness (Nebot Giralt et al., 2017) and poor governance (World Health Organization, 2014).

To address this predicament, five approaches can be differentiated: the excellence approach, the less evil approach*,* the pragmatic approach, the developmental approach, and the legalistic approach.

## Approaches to the quality problem

### The excellence approach

The excellence approach is based on the principle that everybody has the right to the same level of quality health care, irrespectively of contextual constraints: “I will never compromise on quality and will offer the same standard to each patient and each community”. For instance, if a purchaser operates in a country where fully quality-assured antimalarials (i.e. prequalified by WHO, or approved by a stringent regulatory authority) (ACTwatch Group, Newton, Hanson, & Goodman, 2017) are not registered or imported, it is left with two choices: either source them from local manufacturers/suppliers, which will be easier from a logistic point of view, although knowing that the regulator has no (full) capacity to assess and inspect these products to the same rigour as a prequalification/stringent regulator would do; or import them as WHO pre-qualified products, if needed negotiating with the Ministry of Health and facilitating local registration and importation procedures. Noteworthy, if the same purchaser needs to procure first- and second-line antibiotics, or medicines for diabetes or asthma, it cannot count on the guidance of the WHO PQP. Therefore, it should choose between buying locally medicines that are registered locally, or importing products registered by a Stringent Regulatory Authority or equivalent mechanisms, if needed negotiating with the Ministry of Health and facilitating local registration and importation procedures. A purchaser adopting the “excellence approach” will choose in both cases the second option, even if it may require additional efforts, because full quality assurance is preferable to invoking risk margins in safety and efficacy (and, in case of antimalarials and antibiotics, in the possible emergence of resistances). It needs no affirmation, however, that this option is likely to be more complex and costly for local and/or small organizations that do not have a specific budget for importing medicines, and/or lack the expertise needed for preselecting medicines and suppliers, and/or are not in the position for negotiating with the Ministry of Health. In addition, irrespectively of the skills and resources of the purchaser, the complexity would further increase for all those essential medicines that are not covered by the WHO Prequalification Programme, and for which full quality assurance would only be guaranteed by using products registered in countries with stringent regulatory authorities, such as the USA and the countries of the European Union, or by first auditing the suppliers according to stringent standards.

Given the higher cost of the excellence approach *in some circumstances*, this option may be difficult to implement for purchasers with limited resources and negotiating power, and for all medicines not covered by the WHO PQP. However, it remains the first choice in a patient-centred approach, because it avoids exposure of (vulnerable) individuals to *avoidable* health risks.

### The less evil approach

The less evil approach makes a trade-off between the risks in using medicines the quality of which has not been fully verified, and the risk of not getting any drugs at all (for all those in need). For instance, an organization that is facing a stock-out of antimalarial medicines, and that does not have easy access to a supplier of quality-assured antimalarials, may decide to buy a small stock of non-pre-qualified products from a local supplier as the least risky option. The justification for this strategy lies in the fact that, for an acute or life-threatening disease, providing a treatment with possible risks of sub-optimal efficacy is generally thought to entail less risks for the individuals than providing no treatment at all. However, non-quality-assured medicine may also cause direct toxicity, due to mix-ups and impurities, which would result in additional harm. Also, prerequisites to this strategy are that purchasing organizations exhaust reasonable options to keep or restore stocks of pre-qualified medicines, strive to restore the secured supply chain as soon as possible, and correct the stock-planning mistakes that may have caused the stock-out.

The “less evil” approach implies accepting avoidable risks for (vulnerable) patients, and it opens up the risk of persistently ending up in non-secured supply chains. Purchases of non-quality-assured medicines, meant for exceptional circumstances, always imply risks for individual and public health, and should not become routine practice, nor (hopefully) corruptive practice.

### The pragmatic approach

The pragmatic approach is, or should be, based on a risk/benefit assessment at community level: “I prefer a cheaper/easier to procure product versus a more expensive/more complex to procure, but quality-assured one, because I want to treat more people with the same budget”. This might be the case if at a given location a non-quality-assured medicine was cheaper (direct cost) or cheaper/easier to procure (indirect cost) than the corresponding pre-qualified product. If the purchasing organization decided to buy the non-pre-qualified product, its decision might *at first sight* seem justified from the perspective of both the caregivers and the community, because it would allow all those in need to be treated, despite budgetary constraints. However, this decision would imply ignoring some important related issues.

First, manufacturing quality-assured medicines has a price, which is worth being paid. Manufacturers which under-dose the active ingredient, do not check a medicine for impurities or do not carry out any bioequivalence studies, will surely be able to supply very cheap tablets. These will be *administered* to more patients, but *will not treat these patients (*if under-dosed), or they *will harm them* (if contaminated). Comparing the wholesale or retail price of quality-assured and non-quality-assured products, without duly considering compliance with quality, safety and efficacy standards, would be unethical, by ignoring the potential harm to patients and additional costs to the health system and society from poor-quality medicines.

Second, simply assuming that quality-assured medicines will be more expensive may be incorrect, since the price is also linked to manufacturing volumes and market opportunities. In many cases, the WHO PQP has brought international procurement prices of quality-assured medicines down, because manufacturers which invested in WHO prequalification were indirectly rewarded through access to a significant market and, in turn, they had the opportunity for economies of scale.

An organization that chooses to purchase a non-quality-assured medicine purely based on pricing considerations, accepts to structurally take the risk of harming the patients it serves. In addition, by focusing on the short-term “cost” of the medicines, it neglects the more comprehensive and more important “value” of the medicine, i.e. a substandard cheaper medicine will eventually impose higher costs on the health system, because of the failure to cure. If no *short-term* alternative is possible, the purchasing organization should do its best to challenge the conditions that led it to accept a risk for the community it serves, e.g. by improving the stock planning and the supply chain, by negotiating better prices with the pre-qualified supplier, by joining pooled procurement initiatives, by joining advocacy initiatives to call for increased funding of treatment programmes, etc. The inherent risk here is that an ill-informed “pragmatism” ignores the risks linked to lack of quality assurance and accepts a substandard status quo, by focusing on quantitative indicators (how many people are administered a medicine) and on short-term financial gains (cheaper procurement price), with no attention to the quality of care (how many people are *effectively treated*), to the protection and well-being of individuals and the community (how many people are *not harmed*), and to the long-term costs for health systems.

### The legalistic approach

The legalistic approach holds that as long as medicines are perceived to be compliant with applicable domestic laws and requirements, it is justified to procure them for use in a given country, whatever the doubts concerning their quality. The justification for this approach lies in the respect for local governance. This approach, however, falls short in considering the risks that insufficiently regulated environments impose on patients and the community (World Health Organization, 2008, 2010), and may fail to encourage and empower governments, health systems and communities to improve the protection of individual and public health. For example, if a specific antimalarial registered in-country is cheaper than the corresponding WHO prequalified product, or easier to procure, the legalistic approach will lead the purchasing organization to buy the non-pre-qualified product, on grounds of legal acceptability, even if it would be legally and financially possible to procure the prequalified product. Obviously, the *law* cannot discharge purchasing organizations from the *moral* duty to pursue the best possible quality, and this even if, in case of quality accidents, the purchasing organization will not be liable for having selected a poor-quality product.

### The developmental approach

The developmental approach is based on the non-acceptance of an unfair status quo. It considers advocacy for better standards in the future as a must. For example, where fully quality-assured medicines are locally not available (e.g. they are not registered and/or not imported in a given country) or not affordable (for instance, because the innovator product is priced too high for the purchasing power of resource-constrained settings), purchasing organizations will campaign for making the fully quality-assured product(s) available and accessible. This may require explicit campaigning for the primacy of public health over protection of commercial interests and intellectual property rights (Médecins Sans Frontières Access Campaign).

Whenever possible, this should also involve working with partners to encourage and support local manufacturers to come up to prequalification standards and get their products prequalified, while supporting improving national regulatory capacity. These upgraded local manufacturers could then play a more important role at regional level, and the quality-assurance upgrade would be beneficial for all the products in their portfolio, including those that are not currently covered by the WHO Prequalification Programme (PQP). Advocacy will be particularly needed to support those countries that cannot count (anymore) on the support of international donors such as the GFTAM and UNITAID (which have been financing preventive work to sustain quality-assured supplies). For instance, advocacy will also be needed to convince prequalified manufacturers to apply for national registration, and to facilitate national registration of prequalified products using fast-track procedures, i.e. the WHO PQP *Collaborative Procedure* for the accelerated registration of WHO-prequalified products (World Health Organization).

This approach comes at the risk of practical failure due to insufficient skills and/or power to effectively change the current situation. The experience of advocates of access to medicines, however, shows that it is possible to create a powerful advocacy movement which can trigger important changes (Ravinetto et al., 2016).

## Discussion

### Transversal issues

Irrespective of which of the *prevailing* attitudes (excellence, less evil, pragmatic, legalistic, developmental) is consciously or unconsciously adopted by a purchasing organization that is considering compromising on quality assurance, some problems and questions will always arise when such dilemmas are confronted.

First, the question occurs of whether the “institutional attitudes” of purchasers correspond with their “personal attitudes”, i.e. “would I make the same choice if the medicine was for myself, or a family member or friend?”. This is not merely a theoretical question. In many (most) low-income countries, there are pharmacies or distributors specialized in supplying high-cost, quality-assured products for the country’s élites. The choice between “full quality assurance for me” versus “a variable margin of risk for the majority of people” becomes a real dilemma for some purchasers who operate in such environments. This dilemma can be further complicated when purchasing organizations operate outside their own country, which could invoke double standards. For instance, will a European or US-based NGO purchase for local use in LMICs only medical products that meet European Union and/or US standards, or will it consider a different standard, in function of the available budgets (which may have been initially under-estimated) and of local practices? And, if they do so, under which conditions or constraints can they justify this choice?

Second, problems with the quality of medicines may not so much be a problem of the medicine itself (there is, after all, an alternative of better quality), but a problem of attitude, skills and knowledge, and of choices on how to plan and use available resources. Therefore, transparency and accountability concerning compromises on quality assurance and its underlying justifications is the key to raising awareness of the potential *harm* that may be caused by non-quality-assured medicines. Non-specialists may tend to attribute the possibility of harm only to medicines that *have been found* to be of poor quality following *ad hoc* laboratory testing of compliance with quality specifications. Nonetheless, the potential harm is in the first instance an attribute of *non-quality-assured medicines*, that is those that have been approved and made available without going through a stringent regulatory evaluation. Laboratory testing is, together with other regulatory activities such as dossier assessment and inspections, a component of stringent regulatory evaluation, but if taken in isolation it is not sufficient to rule out all quality problems. For instance, it will miss the presence of toxic impurities that may unexpectedly come into the medicine by mix-up during manufacturing, or due to the use of active ingredients of unknown synthesis, etc. (World Health Organization, 2003). Ignoring the importance of these distinctions can lead many stakeholders and policy-makers to compromise on quality without awareness of the potential for harm.

Third, the question arises as to whether an international purchasing organization (or a donor) will make the same or a different choice, in different contexts/countries. In general, procurement strategies may vary depending on different local constraints, e.g. different local patterns of registration or pricing of quality-assured medical products, and different regulatory and legal requirements (such as importation rules). Thus, it is important that any decision to temporarily compromise on quality assurance is grounded in (and justified upon) clear ethical values and criteria. For instance, a pragmatic decision to buy a product that may not necessarily comply with all quality assurance standards may be acceptable, if based on a reasoned risk/benefit assessment and accompanied by risk-mitigating measures, in a country and circumstances with no better options, while it would be unethical to purchase the same product in a context in which better, affordable options are available. It is also important that what “no better options” means is clearly defined on a case-by-case basis; and it must be avoided that the alternative options (to compromise) that are “difficult but feasible” are straightforwardly classified as “unfeasible”.

### An ethics approach to dilemmas in pharmaceutical purchase

Poor-quality medicines may cause serious harm, including death or permanent disability. Any time a non-quality-assured medicine is purchased, it is impossible to accurately foresee if/which quality problems may appear; however “reasonable” the risk may appear, the purchasers and users will never be fully “in control” of such risk. Therefore, there is a moral obligation that under any circumstances, due attempts should be made to apply the best possible quality assurance methods and standards, in order not to expose patients to possible harm from non-quality-assured medicines or unsecured supply channels. When having to deliberate on possible compromises on quality assurance, purchasers and organizations should be aware that they are dealing with conflicting values, such as immediate availability (*beneficence*) to all those in need (*justice*), the duty to avoid preventable harm (*beneficence* and *non-maleficence*), and the duty to avoid using different standards across different populations (*justice* again). Most likely, in making such a decision they might – often tacitly – refer to general ethical principles, personal and/or institutional interests and values, and concerns about the health and well-being of the target population. We contend that making the reasoning behind purchasing decisions explicit will improve the transparency and the accountability of the process, which eventually benefits decision-making itself. Some of the questions they should address for making their ethical reflection explicitly grounded in values, are as follows:

- Why is a (fully) quality-assured medicine not a viable option under the current circumstances?
- Is there sufficient and adequate expertise in-house to make a balanced quality risk assessment? If not, where may we seek qualified advice?
- What is the proposed compromised scenario? What are the risks for the individual patients and for their community from the non (fully) quality-assured medicine?
- Are the envisaged risks ethically justifiable? Why? And who is going to take the final decision?
- Would the same compromise be considered acceptable in a different context (in particular, in the country of the purchasing or funding organization)? Why?
- Is the compromise envisaged only in the short term (e.g. in order to confront an acute situation such as a stock-out or an unplanned emergency) or in the long term? Why?
- Will any actions be put in place to mitigate the risks related to the envisaged compromise (e.g. advocacy plans)?
- Who is entitled, in the organization, to make a decision on this matter?

As above, under all circumstances due attempts should be made to apply the best possible quality assurance methods and standards. If, after completion of this exercise, the proposed compromise is considered to be acceptable *temporarily* and *under the given circumstances*, the organization should be able to describe the kind of compromise, the “quality risk assessment”, and the decision-making process, and to explicitly justify the choice vis-à-vis the relevant stakeholders, and in particular the concerned community and their gatekeepers. Engaging with the communities and, when possible, with national regulators and policy-makers, is of paramount importance, to ensure an appropriate balance between the duty to cure in the best possible way, and the contextualization of the quality risk assessment.

## Conclusions

Access to quality-assured medicines is an essential prerequisite for high-quality health care. Unfortunately, the problem of poor-quality medicines is well documented in resource-limited settings, and procedural approaches are not (yet) sufficient to resolve it. This results in (the risk of) *harm* to individual and public health. Manufacturers and distributors selling their products in and for poorly-regulated markets, are not *compelled* by stringent regulatory and legal mechanisms to systematically implement good practices in pharmaceutical production and distribution. It is noteworthy that similar technical and ethical dilemmas also arise for other medical products, such as vaccines, in vitro diagnostics and medical devices.

We believe that, in the absence of regulatory mechanisms that *compel* the systematic implementation of good practices in pharmaceutical production, and in the absence of market incentives to do so, there is an *ethical duty* for pharmaceutical suppliers to minimize or eliminate avoidable risks due to poor-quality medicines, by systematically implementing good practices; and that failure to do so should be seen as a violation of the ethical principles of beneficence and non-maleficence.

Purchasers will often face concrete dilemmas about possible compromises vis-à-vis the internationally agreed quality standards; and since the harm due to poor quality medical products often goes undetected (except in clustered cases of acute toxicity), they may be tempted to under-estimate the likelihood that patients and communities will be harmed.

We contend that empirical bioethics should acknowledge the existence of these dilemmas in public health, and help to build a normative approach to dealing with them. Ethics approaches must be explored and put on the agenda, e.g. by integrating them in the main international codes and declarations. As a starting point, some questions should be addressed:

- What are the role and ethical responsibility of governments and regulators, both in the country(ies) of manufacture and in the country(ies) of (transit and) destination?
- What are the role and ethical responsibility of manufactures and distributors?
- What are the role and ethical responsibility of prescribers and healthcare-providers?
- What are the role and ethical responsibility of health funding agencies?
- Which principles should guide the ethical reasoning in this domain?

We would also suggest that training programmes in regulatory affairs, pharmacy practice, quality assurance, pharmaceutical supply and procurement, public health programmes with supply component, health economics, etc. should always include principles of the quality assurance ethical dimension, especially for those operating in and for resource-constrained settings. In addition, stakeholders confronted with ethical dilemmas in pharmaceutical procurement should consider “going public” and advocate for universal access to quality-assured medicines.

We would also recommend that an international group of experts in quality assurance/regulatory affairs and health ethicists should be set up to take up this topic and formulate a Guide to Ethical Principles of Quality Assurance of Medical Products, preferably covering all groups: medicines, including vaccines and other biologicals, and medical devices.
